# Atmosphere-Ocean Coupling Effect on Intense Tropical Cyclone Distribution and its Future Change with 60 km-AOGCM

**DOI:** 10.1038/srep29800

**Published:** 2016-07-15

**Authors:** Tomomichi Ogata, Ryo Mizuta, Yukimasa Adachi, Hiroyuki Murakami, Tomoaki Ose

**Affiliations:** 1 Faculty of Life and Environmental Sciences, University of Tsukuba, Japan; 2 Meteorological Research Institute, Tsukuba, Japan; 3 Japan Meteorological Agency, Tokyo, Japan; 4 Geophysical Fluid Dynamics Laboratory, Princeton, USA

## Abstract

Atmosphere-ocean coupling effect on the frequency distribution of tropical cyclones (TCs) and its future change is studied using an atmosphere and ocean coupled general circulation model (AOGCM). In the present climate simulation, the atmosphere-ocean coupling in the AOGCM improves biases in the AGCM such as the poleward shift of the maximum of intense TC distribution in the Northern Hemisphere and too many intense TCs in the Southern Hemisphere. Particularly, subsurface cold water plays a key role to reduce these AGCM biases of intense TC distribution. Besides, the future change of intense TC distribution is significantly different between AOGCM and AGCM despite the same monthly SST. In the north Atlantic, subsurface warming causes larger increase in frequency of intense TCs in AOGCM than that in AGCM. Such subsurface warming in AOGCM also acts to alter large decrease of intense TC in AGCM to no significant change in AOGCM over the southwestern Indian Ocean. These results suggest that atmosphere-ocean coupling characterized by subsurface oceanic structure is responsible for more realistic intense TC distribution in the current climate simulation and gives significant impacts on its future projection.

Previous studies have suggested that the frequency of extreme events, such as heavy precipitation over East Asia^1^, intense extratropical cyclones^2^, and heat waves^3^, will increase in the future. Tropical cyclones (TCs) are an important category of such extreme events, as they can cause substantial impacts on populated areas, in the form of strong winds and precipitation. Over East Asia and North America, in particular, TCs commonly lead to social and economic disasters where they make landfall. There is, therefore, considerable need for accurate regional projections of future TC activity^4^,^5^,^6^,^7^. Recent studies have found agreement in projections of a global reduction in TC number and an increase in mean TC intensity^8^,^9^,^10^,^11^,^12^,^13^ caused by a slowdown of the tropical circulation^14^,^15^. However, projected changes in individual basins remain uncertain^10^,^16^,^17^. Since the 1990s, advances in computational resources have made high-resolution general circulation model (GCM) simulations possible. For example, the intensities of TCs simulated by the 20 km resolution atmosphere-only general circulation model (AGCM; MRI-AGCM3.2S) were much more realistic than results from the previous version of the model (MRI-AGCM3.1S)^18^. Using these models, a previous study^19^ found that TC frequency in the future climate decreased in both models over the Northwest Pacific (NWP), while projected frequency changes over the North Atlantic (NATL) were different between MRI-AGCM3.1S and MRI-AGCM3.2S, making the true situation unclear. However, in the present climate simulation in MRI-AGCM3.2S, a northward bias remains in the spatial distribution of the most extreme TCs (those with a maximum wind speed of more than 70 ms^−1^, known as Category 5 or C5) compared with observations^18^.

Previous studies have investigated the possible causes of such biases in intense TC distribution. Some have noted the importance of rapid and large TC-induced SST cooling that occurs through changes in the ocean subsurface^20^,^21^,^22^, although this explanation is not relevant to AGCM simulations using prescribed SST. In the Northern Hemisphere (NH), the underlying subtropical mode water (STMW^23^,^24^,^25^) raises the thermocline at around 20–30°N, making the oceanic mixed layer shallower than at 10–20°N. This shallow mixed layer north of 20°N may suppress TC intensification through SST cooling. To test whether such an atmosphere–ocean coupling process can reduce the northward bias seen in the TC distribution in AGCMs using prescribed SST, coupling with an oceanic GCM (OGCM) is needed. A previous study^26^ investigated the importance of atmosphere–ocean coupling in assessing the distribution of intense TCs over the NWP using a 60 km resolution coupled atmosphere–ocean general circulation model (AOGCM). In this work, we extend the approach to study the effect of atmosphere–ocean coupling on intense TCs in other ocean basins, and their projected future changes.

## Results

**Figure 1:**
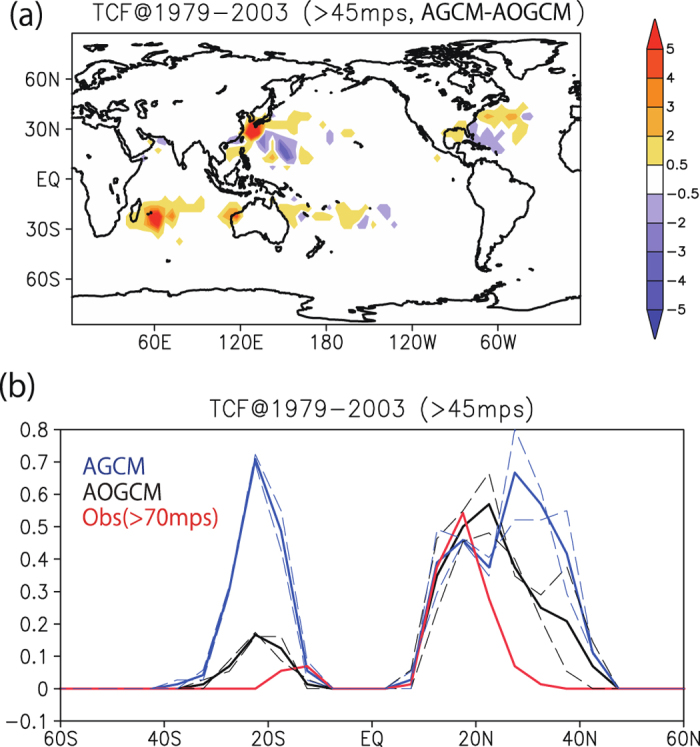
Air–sea coupling effect on the present-day intense TC frequency. (**a**) Difference in the intense TC (maximum wind speed >45 ms^−1^) frequency between the AGCM and AOGCM simulations, in 5° × 5° bins for 1979–2003 (units of (25 yr)^−1^). (**b**) Zonal average intense TCF (in 5° × 5° bins; units of (25 yr)^−1^). The AOGCM (AGCM) results are shown in black (blue). Dashed lines show subsamples of the full period (1979–1990 and 1991–2003). Observed extreme (C5: maximum wind speed >70 ms^−1^) TCF is shown in red. All plots and maps are generated by GrADS version 2.0.2 (http://cola.gmu.edu/grads/).

First, we measure the atmosphere–ocean coupling effect by comparing the TC climatology between AGCM and AOGCM simulations. Figure 1a shows the intense TC frequency (TCF) difference between the two simulations, in 5° × 5° bins during 1979–2003 [see a previous study^18^ for details of the TC tracking method]. The 60 km resolution of the AGCM is too coarse to realistically capture the frequency of C4 or C5 extreme TCs as seen in a 20 km AGCM^18^. Therefore, we choose TCs stronger than C3 (maximum wind speed >45 ms^−1^) as ‘intense’ for this study. Although the 60 km AGCM has a quantitative bias in the TC intensity distribution due to its coarse resolution, the 20 km and 60 km AGCMs share common features in their northward bias in the TC intensity peak^19^.

The present study focuses on whether atmosphere–ocean coupling in the 60 km AOGCM can reduce the northward bias qualitatively compared with the 60 km AGCM. A positive TCF difference (Fig. 1a) around 20–30°N shows that the AGCM tends to simulate intense TCs more frequently than does the AOGCM, and that the northward bias can be seen not only in the NWP^26^ but also in the NATL and the Southern Hemisphere (SH). This result indicates that the simulation of intense TCF is affected by the atmosphere–ocean coupling present in the AOGCM.

Figure 1b shows the meridional distribution of zonally averaged intense TCF during 1979–2003. In the AOGCM (black lines), the intense TCF peak is located around 15–25°N, while the AGCM (blue lines) has a NH peak around 30°N. In the SH, an intense TCF peak around 25°S is greatly reduced in the AOGCM. Similar TCF distributions in different periods (1979–1990 and 1991–2003) suggest that the increase in intense TCF in the AGCM is robust around 25–35°N and 15–30°S. Area averages for individual basins are listed in Table 1. In the NWP and the NATL, a robust decrease in the AOGCM compared with the AGCM is found around 25–40°N (45% of AGCM in the NWP, and 48% of AGCM in the NATL). Decrease in AOGCM (11% of AGCM) in the southwestern Indian Ocean (SWIO) is also robust.

For comparison, observed intense TCF (using only C5 TCs; i.e., those with maximum wind speed of more than 70 ms^−1^) during 1979–2003 is also shown in Fig. 1b. The peak in observed intense TCF is found around 10–20°N, and TCF declines rapidly north of 20°N. Comparison between the observed and AOGCM intense TCFs indicates that observed intense TCs induce greater SST cooling (cold-wake) via stronger TC-induced mixing, resulting in a more rapidly decreasing TCF distribution with increasing latitude. In the SH, the observed intense TCF is much smaller than in the NH. In contrast to the AGCM, the AOGCM captures this north–south asymmetry in the TCF. Overall, the intense TCF in the AOGCM exhibits more realistic features (e.g., the shift in the NH peak and the north–south asymmetry) than does that in the AGCM. These results indicate that atmosphere–ocean coupling is crucial for realistic simulations of intense TC distribution.

**Figure 2:**
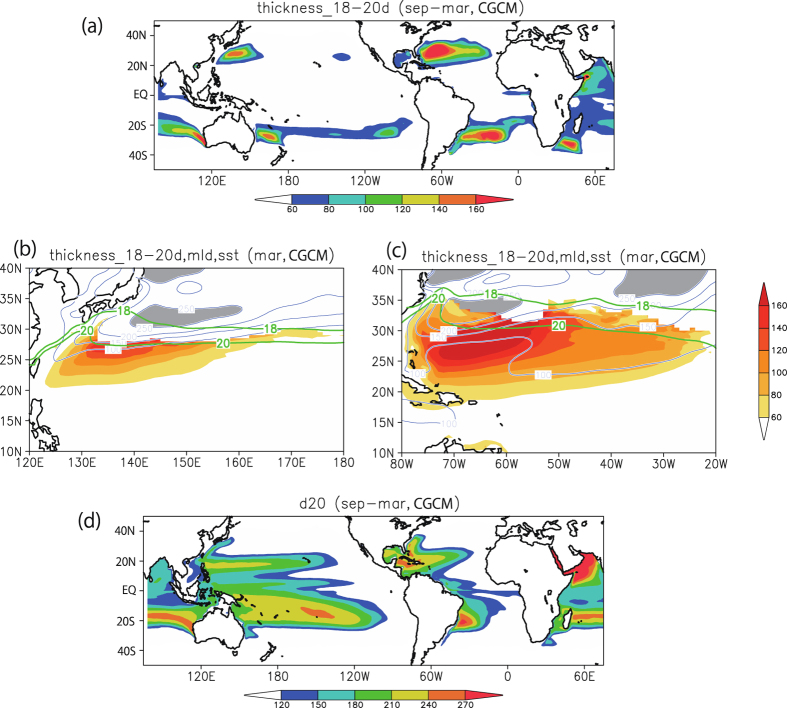
Subsurface ocean state in the AOGCM. (**a**) Horizontal distribution of AOGCM-simulated STMW (defined as 18–20 °C water thickness, units of m) in late summer (September in the NH, March in the SH). (**b**) Same as (**a**), but for late winter (March) in the NWP and (**c**) in the NATL. (**d**) Same as (**a**), but for the 20 °C isotherm depth in late summer. In (**b**,**c**), MLD (SST) is shown by blue (green) contours, and MLDs of greater than 250 m are shaded in grey. All plots and maps are generated by GrADS version 2.0.2 (http://cola.gmu.edu/grads/).

We now examine the behaviour of the subsurface ocean in the AOGCM. Figure 2a shows the subsurface 18–20 °C water thickness, as a measure of the STMW, in late summer in each hemisphere. In the NH, thick STMW is present around 20–35°N, which raises the thermocline (Fig. 2b,c). The STMW is formed during winter and spring (Fig. 2b,c). Thick, uniform mode water can form along the mixed layer depth (MLD) front, and the SST at the MLD front determines the properties of the STMW. In summer, the shallow seasonal thermocline produced via surface heat flux prevents the subduction of the STMW, but thick and uniform 18–20 °C water remains (Fig. 2a). On the other hand, in the SH the contribution of thick 18–20 °C water appears to be relatively weak. In the SWIO, the 18–20 °C water thickness maximum cannot be clearly seen (Fig. 2a). Instead, a shallow 20 °C isotherm appears around 5–15°S over the SWIO (Fig. 2d). Such shallow isotherm is a result of Ekman pumping and oceanic Rossby waves^27^,^28^, and may contribute to the less intense TCF in the SH.

**Figure 3:**
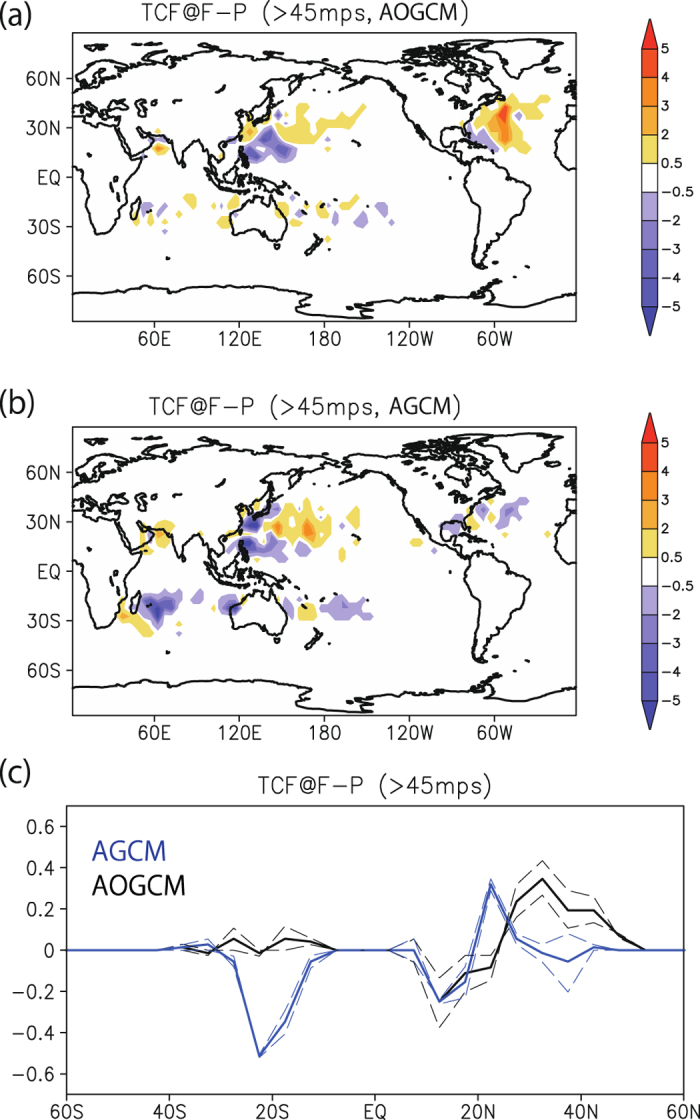
Differences in future changes in intense TC frequency between the AOGCM and the AGCM. (**a**) Change in intense TCF between current and future climate simulations, in 5° × 5° bins, in the AOGCM (units of (25 yr)^−1^). (**b**) Same as (**a**), but for the AGCM. (**c**) Zonal average of intense TCF (in 5° × 5° bins; units of (25 yr)^−1^), for the AOGCM (black) and the AGCM (blue). Dashed lines show subsamples of the full period (2075–2086 minus 1979–1990, and 2087–2099 minus 1991–2003). All plots and maps are generated by GrADS version 2.0.2 (http://cola.gmu.edu/grads/).

Next, the effect of atmosphere–ocean coupling on the future climatology of intense TCs is investigated by comparing AGCM and AOGCM future climate simulations. A difference in the projected change in intense TCF can be seen over the NATL and the SWIO (Fig. 3a,b). In the NATL, the AOGCM shows a clear increase in intense TCF in the future, while such an increase is unclear in the AGCM. In the SWIO, the AGCM shows a clear decrease in intense TCF in the future, while such a decrease is unclear in the AOGCM. In terms of the zonal average (Fig. 3c), both AGCM and AOGCM show a small decrease in intense TCF around 15°N, and that AGCM shows an increase in TCF around 20°N while AOGCM shows an increase around 30–40°N. In the SH, the AGCM shows a large decrease in the future around 25°S, while the AOGCM shows a weak increase. Differences between the models in terms of projected changes in intense TCF are not sensitive to different subsample periods (Fig. 3c), indicating that the differences around 20–50°N and 10–30°S are robust.

Area averages in individual basins are provided in Table 2. An increase in the NATL in the AOGCM (178%) and a decrease in the SWIO in the AGCM (62%) are robust, while changes in the NWP are not robust, due to large internal variability. According to observational analysis^29^ during 1970–2004, the number of the Category 4-5 TCs increases almost all over the basins. However, a recent observational study^30^ showed that the trends of the number of the Category 4–5 TCs during a more reliable period of 1990–2014 are insignificant all over the basins including the global average. Small SST warming of about 0.5 °C in observation (3 °C in our study) may explain the difference between the observational analysis and the future projections here^9^.

**Figure 4:**
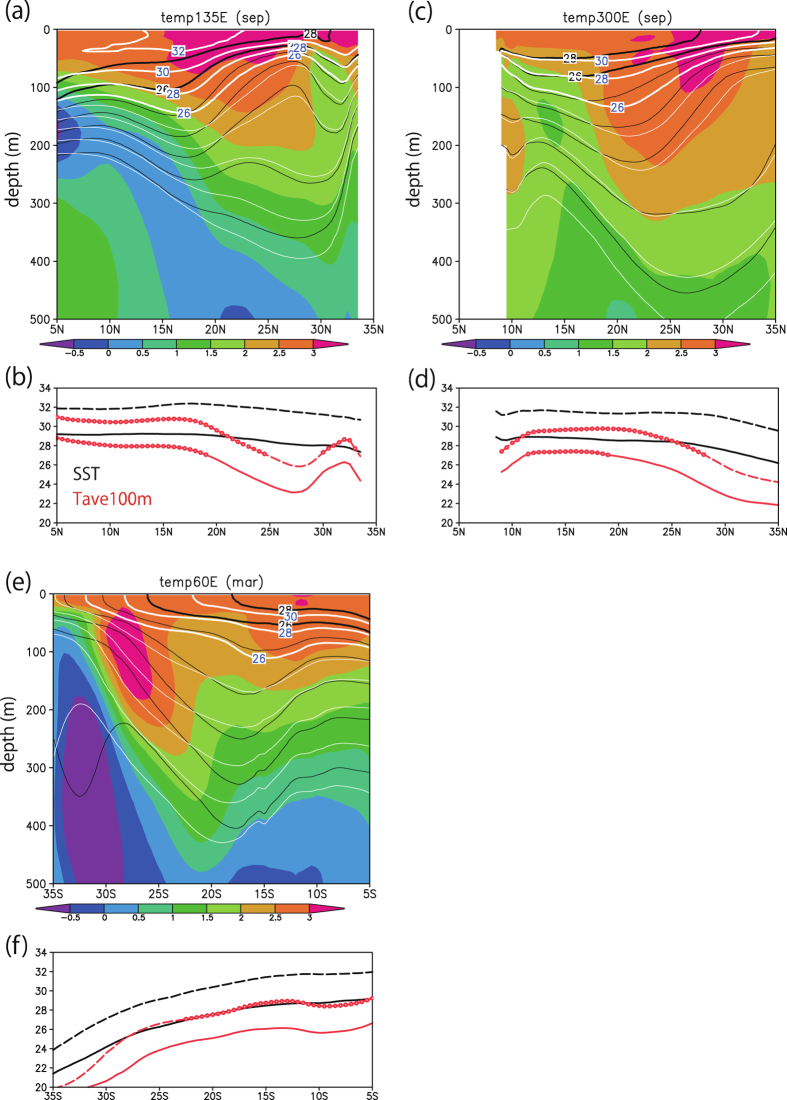
Future changes in the subsurface ocean. (**a**) Latitude–depth cross-sections of ocean temperature in the present (black contours) and future (white contours) climates, and the change (shading), and (**b**) meridional distributions of SST (black, in °C) and Tav100 (red, in °C) in the present (solid lines) and future (dashed lines) climates in September at 135°E. (**c,d**) Same as (**a,b**), but for 60°W in September. (**e,f**) Same as (**a,b**), but for 60°E in March. Open circles in (**b,d,f**) mark areas with temperature above 27 °C. All plots and maps are generated by GrADS version 2.0.2 (http://cola.gmu.edu/grads/).

In addition to SST warming, the AOGCM shows a subsurface ocean warming, but the meridional structure differs between the surface and subsurface (Fig. 4). Previous studies have shown that vertically averaged ocean temperature is a good measure of mixing by intense TCs^20^,^21^. In this study, temperature averaged over the upper 100 m (Tav100) is used to represent the upper portion of the ocean that interacts with TCs and defines ocean heat content available for TC intensification. Tav100 is especially modified by the ocean response to TCs by mixing and upwelling^20^,^21^. In the NATL, SST is fairly uniform in the present climate simulation and exceeds 27 °C almost everywhere, but Tav100 exceeds 27 °C around 10–20°N only. These cold subsurface conditions (also seen in Fig. 2) in the NATL contribute to the lower intense TCF in the AOGCM compared with the AGCM (Fig. 1). In the future climate simulation, the region in which Tav100 exceeds 27 °C extends to 30°N (Fig. 4c,d). Compared with the other basins, the deeper future subsurface warming weakens the subsurface impact on intense TCs, meaning that intense TCF change in the AOGCM is greater than that in the AGCM (Fig. 3). In the SWIO, Tav100 does not exceed 27 °C anywhere in the present climate (Fig. 4e,f). The cold subsurface (also seen in Fig. 2) contributes to the decrease in TCF in the AOGCM compared with the AGCM (Fig. 1). In the future climate, a region in which Tav100 exceeds 27 °C appears north of 25°S. This subsurface warming in the SWIO weakens the subsurface impact on intense TCs, leading to a weak increase in TCF in the AOGCM, whereas a large decrease is simulated by the AGCM (Fig. 3). In the NWP, a cold area (Tav100 below 27 °C) remains around 25–30°N in the future climate (Fig. 4a,b). Furthermore, compared with the NATL, subsurface warming is shallower in the NWP (2.5 °C warming extends to 200 m depth in the NATL, but only to 150 m in the NWP). This shallow subsurface warming in the NWP may be responsible for the relatively small difference in TCF change between the AOGCM and the AGCM. It should be noted that the surface and subsurface warming are robust over the two subsampled periods (Supplementary Figure S1). A map of climatology and projected future changes in SST and temperature averaged over the upper 100 m and 200 m (Supplementary Figure S2) shows that deep warming reaching to 200 m (about 2.5–3 °C in magnitude) can be seen around 20–30°N. In the NATL, compared with the NWP, there is a larger meridional separation between the 24 °C (equal to about 27 °C in the future climate) and 27 °C contours. Thus, subsurface thermodynamic conditions favourable to TC development (temperature above 27 °C) extend farther northward in the NATL than in the NWP. Overall, differences in the projected future changes in TCF can be attributed to the biases in the present-day simulations^31^ and to the future changes in subsurface thermodynamic conditions. This result highlights the fact that the ability to accurately simulate the present-day intense TCF is also important for the projection of future changes in intense TCF.

## Summary and Discussion

The effect of atmosphere–ocean coupling on the frequency distribution of intense TCs and their future change has been studied using atmosphere-only (AGCM) and coupled (AOGCM) model experiments. The AOGCM displays smaller biases in TC distribution than does the AGCM, due to regionally varying atmosphere–ocean coupling. In the NH the peak in the distribution of intense TCs shifts equatorward in the AOGCM compared with the AGCM. The frequency of intense TCs in the SH decreases in the AOGCM, which better captures the observed north–south asymmetry in intense TC frequency. A reduction on the poleward flank of the intense TC meridional distribution is attributed to a cold subsurface ocean.

Projected future changes in the TC distribution are different in the AOGCM and the AGCM, despite the models having the same monthly mean SST in both the present and future climates. In the NATL, subsurface warming (exceeding 26–27 °C) in the AOGCM causes a large increase in intense TCs. Subsurface warming also acts to alter the projected change in the SWIO from a large decrease in intense TCs in the AGCM to an insignificant change in the AOGCM. These results suggest that atmosphere–ocean coupling, characterized by the subsurface ocean structure, leads to significant qualitative differences in the projected changes in intense TCs between the AGCM and the AOGCM.

We focused on the effect of atmosphere–ocean coupling on intense TCs only. Considering all TCs, the spatial pattern of changes in TCF is similar in both the AOGCM and the AGCM (Supplementary Figure S3), and is consistent with previous studies^18^,^19^. For example, an overall decrease in TCF is found in the SH in both models although a decrease in the AOGCM is insignificant in the SWIO, and significant TCF decreases in the NWP (west of around 160°E) and the eastern Pacific (east of around 120°W), and an increase in the central Pacific^32^ (around 120–180°W) are common features between the AOGCM and the AGCM. On the other hand, the TCF is decreased in the western North Atlantic by a similar amount in both models, but in the eastern north Atlantic it is increased in the AOGCM and decreased in the AGCM. It should be noted that the spatial pattern of TCF change in the NATL in the AOGCM is similar to that found in previous studies^33^,^34^. In the eastern Atlantic, the climatological 20 °C isotherm is relatively shallow, indicating that strong atmosphere–ocean coupling may be active in this region (not shown). A series of MRI-AGCM3.2 experiments^9^ (Supplementary Figure S4) also shows TCF decreases over the SH and in the NWP, and an increase in the central Pacific, which are robust with respect to the choice of future emissions scenario (CMIP3-A1B or CMIP5-RCP8.5), prescribed SST pattern^35^, and convection scheme. These features are also consistent with previous studies^8^,^9^,^10^,^11^,^12^,^13^. Other existing literature^14^,^15^ suggests that a slowdown of tropical circulation due to increase of vertical dry stability by the global warming can explain global TC decrease generally. For this reason, it is natural to consider that intense TCF also decreases in the SH and NWP under the global warming as well as weak TCF, though vertical wind shear is also important for regional uncertainty such as NATL^10^,^16^,^17^. On the other hand, TC intensity and intense/extreme TCF changes may include different aspects from case of all-TCF. In the former case, increase of TC intensity by local SST or subsurface ocean warming^36^ also becomes important. Our results of future experiments on air-sea coupling effects seem to support this aspect.

To obtain more realistic extreme TC distribution, recent studies suggest that an AOGCM with resolution of 20 km or finer is needed^37^. Our study has clarified the atmosphere–ocean coupling effect in a 60 km resolution AOGCM, but other recent studies indicate that further experiments with finer-resolution (e.g., 20 km) AOGCMs are needed to measure the sensitivity of the effect to resolution. As a next step, a 20 km resolution AOGCM experiment, producing more realistic TC intensity distribution, is necessary to more accurately measure the atmosphere–ocean coupling effect. Furthermore, a high-resolution ocean, with resolution of 20 km or finer, is needed to adequately model mid-latitude SST fronts and narrow coastal currents^38^,^39^,^40^. A high-resolution ocean may also be important to more adequately simulate TC life-cycles^41^,^42^. Finally, TC activity may also have an impact on mean subsurface thermal structure and meridional heat transport^43^. Investigation of the role of TCs in controlling climate should be continued in order to improve climate modelling.

## Data and Models

The AOGCM used in this study consists of an atmospheric global model of MRI-AGCM3.2H^45^, with a 60 km horizontal resolution and 64 vertical levels (TL319L64), and an oceanic model of MRI.COM3^46^, with a tripolar grid with 1° zonal and 0.5° meridional resolution, and 50 vertical levels. To adequately resolve the upper ocean structure, 10 levels (15 levels) are located in the upper 100 m (200 m). The model is the same as that used in a previous study^26^.

Flux adjustment is often used in coupled GCMs to ensure realistic model simulations^47^. To simulate sub-seasonal atmosphere–ocean interaction while reproducing observed SST variability on the seasonal-to-interannual timescale in the present climate, monthly mean flux adjustment was performed in the AOGCM. This flux adjustment process was described in a previous study^26^. Here we refer to a simulation using the MRI-AGCM3.2H atmospheric model with prescribed SST from the AOGCM as the ‘AGCM’ simulation, while the atmosphere–ocean coupled simulation is referred to as the ‘AOGCM’ simulation. Each model was integrated from January 1979 to December 2003 to produce ‘present’ climate simulations^26^. Similarly, ‘future’ climate simulations were performed from January 2075 to December 2099 by adding future changes of boundary conditions (e.g., SST and sea ice) taken as the ensemble mean output of 28 CMIP5 models run under the RCP8.5 scenario^35^ to their present (1979–2003) observed values.

## Additional Information

**How to cite this article**: Ogata, T. *et al*. Atmosphere-Ocean Coupling Effect on Intense Tropical Cyclone Distribution and its Future Change with 60 km-AOGCM. *Sci. Rep.* **6**, 29800; doi: 10.1038/srep29800 (2016).

## Supplementary Material

Supplementary Information

## Figures and Tables

**Table 1:** Area averages of intense TC frequency (maximum wind speed >45 ms^−1^) in individual basins during 1979–2003 (units of (25 yr)^−1^).

	60 km AOGCM	60 km AGCM
SWIO (40–80°E, 15–30°S)	**0.213**	**1.876**
NWPs (120–160°E, 10–25°N)	3.537	2.918
NWPn (120–160°E, 25–40°N)	**0.780**	**1.705**
ATLs (40–70°W, 10–25°N)	0.219	0.053
ATLn (40–70°W, 25–40°N)	**0.892**	**1.842**

Bold characters indicate that the differences between the AOGCM and the AGCM have the same sign for comparisons during 1979–1990 and 1991–2003.

**Table 2:** Area averages of intense TC frequency (maximum wind speed >45 m/s) change (future minus present) (units of (25 yr)^−1^).

	60 km AOGCM	60 km AGCM
SWIO (40–80°E, 15–35°S)	−0.004	−**1.233**
NWPs (120–150°E, 10–25°N)	−1.950	−1.695
NWPn (150–180°E, 20–35°N)	0.952	1.459
ATL (30–70°W, 15–45°N)	**1.053**	−0.234

Bold characters indicate changes that satisfy two conditions: (1) the change is of the same sign for differences between 1979–1990 and 2075–2086, and between 1991–2003 and 2087–2099, and (2) the changes in the AOGCM and the AGCM are significantly different.
