# Supplementary material for: Atmosphere-Ocean Coupling Effect on Intense Tropical Cyclone Distribution and its Future Change with 60 km-AOGCM
Source: Sci Rep. 2016 Jul 15;6:29800. doi: 10.1038/srep29800 (PMC4945927; doi:10.1038/srep29800)
Supplement: Supplementary Information [file srep29800-s1.pdf]

Atmosphere-Ocean Coupling Effect on Intense Tropical Cyclone Distribution and its Future Change with 60km-AOGCM

Tomomichi Ogata[1],

Ryo Mizuta[2], Yukimasa Adachi[2, 3], Hiroyuki Murakami[4],

Tomoaki Ose[2]

[1]Faculty of Life and Environmental Sciences, University of Tsukuba, Japan

[2]Meteorological Research Institute, Tsukuba, Japan

[3]Japan Meteorological Agency, Tokyo, Japan

[4]Geophysical Fluid Dynamics Laboratry, Princeton, USA

Submitted to Scientific Reports

May 2016

Corresponding Author: T. Ogata, Faculty of Life and Environmental Sciences, University of Tsukuba, 1-1-1 Tennoudai, Tsukuba, Ibaraki 305-8572, Japan (ogata.tomomichi.ga@u.tsukuba.ac.jp)

(Supplementary Information)

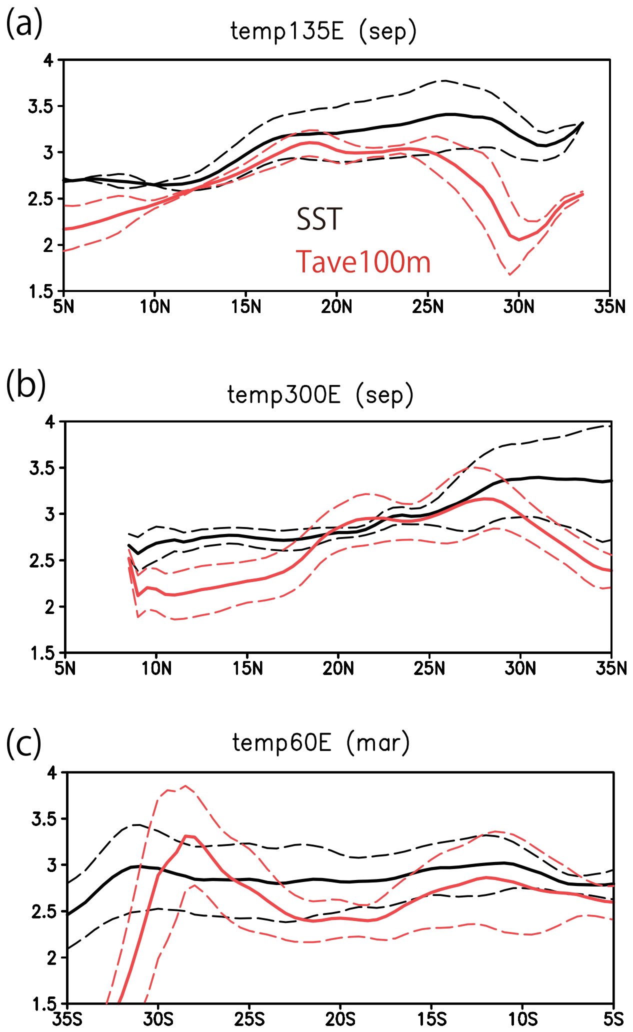

Figure S1: (a) Change of SST (black, unit °C) and Tav100 (red) (lower panel) between future and current climate simulations in September at 135°E. (b) Same as (a) but for 60°W in September. (c) Same as (a) but for 60°E in March. Broken lines show subsamples of 12-yr average (1979-1990, 2075-2086) and 13-yr average (1991-2003, 2087-2099). All plots and maps are generated by GrADS version 2.0.2 (http://cola.gmu.edu/grads/).

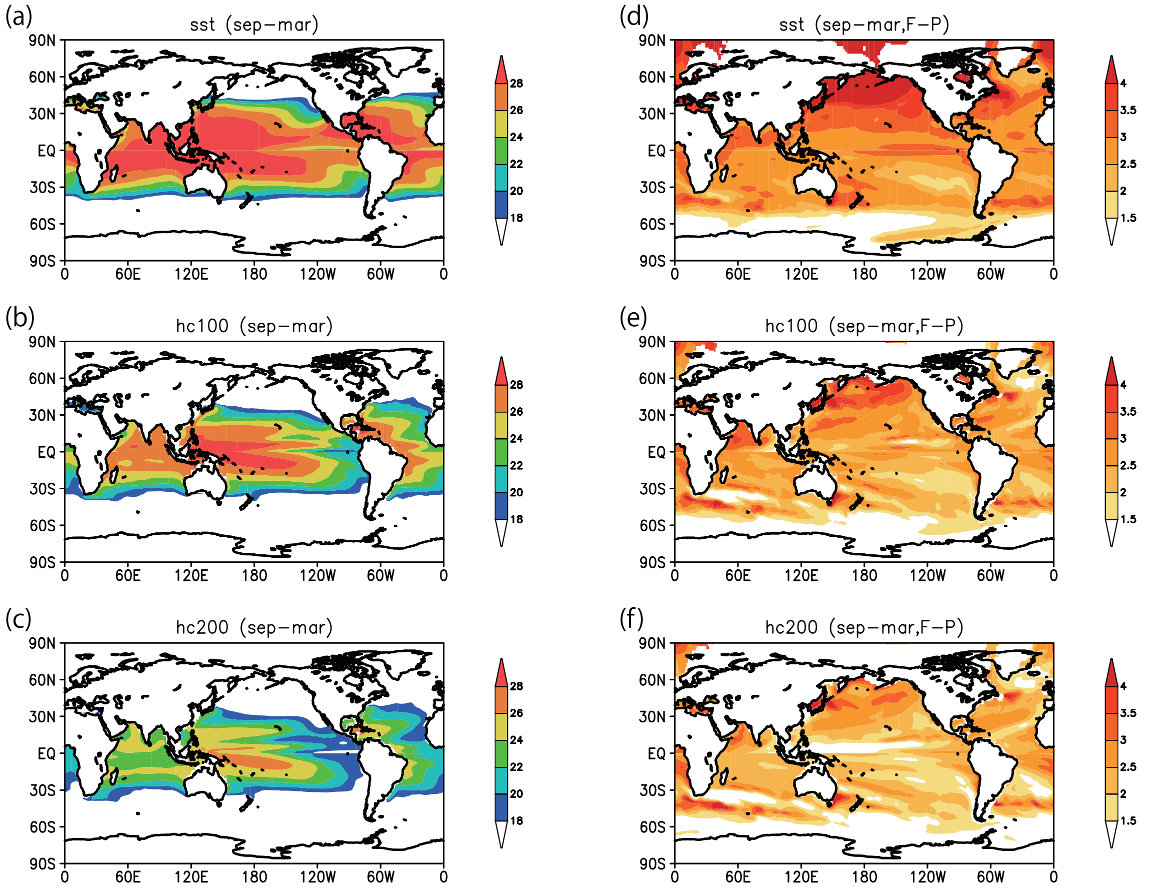

Figure S2: (a) SST climatology (unit °C) in 60km-AOGCM simulated present climate during September in NH (March in SH). (b) Same as (a) but for vertical averaged temperature in the upper 100m. (c) Same as (b) but for in the upper 200m. (d)-(f) are same as (a)-(c) but for changes between future and present climate simulations. In (d)-(f), areas of same signs between subsamples of 12-yr average (1979-1990, 2075-2086) and 13-yr average (1991-2003, 2087-2099) are only shaded. All plots and maps are generated by GrADS version 2.0.2 (http://cola.gmu.edu/grads/).

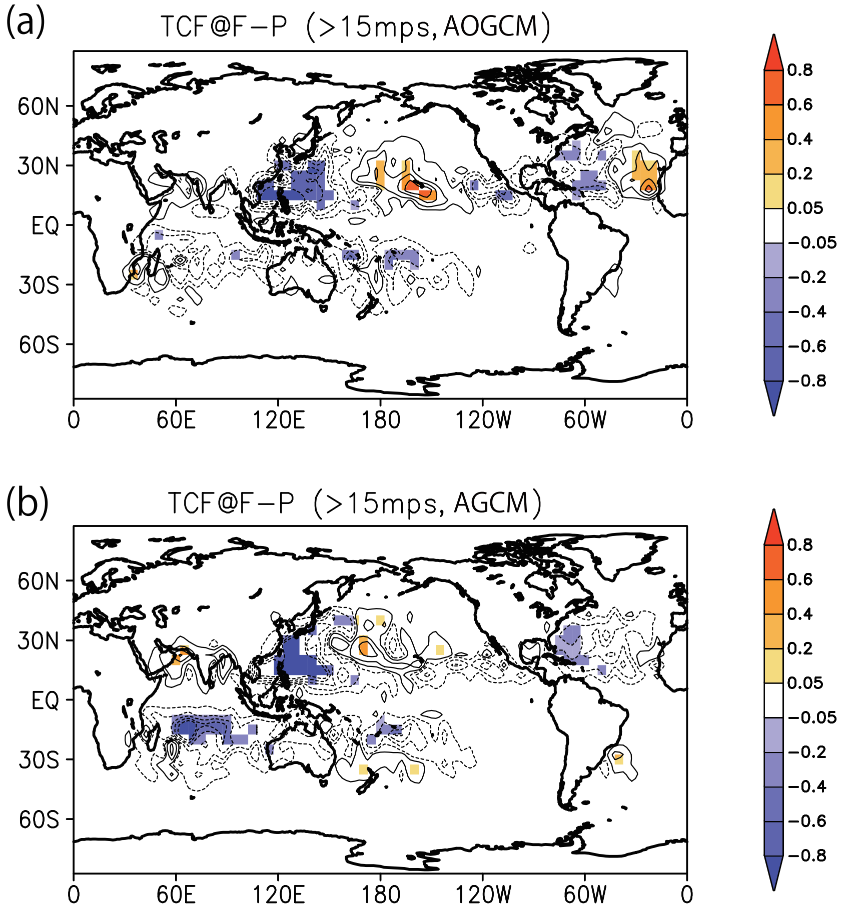

Figure S3: (a) Same as Fig. 3a but for all TCs in AOGCM (unit numbers/yr). (b) Same as (a) but for AGCM. Areas of same signs between subsamples of 12-yr average (1979-1990, 2075-2086) and 13-yr average (1991-2003, 2087-2099) are only shaded. All plots and maps are generated by GrADS version 2.0.2 (http://cola.gmu.edu/grads/).

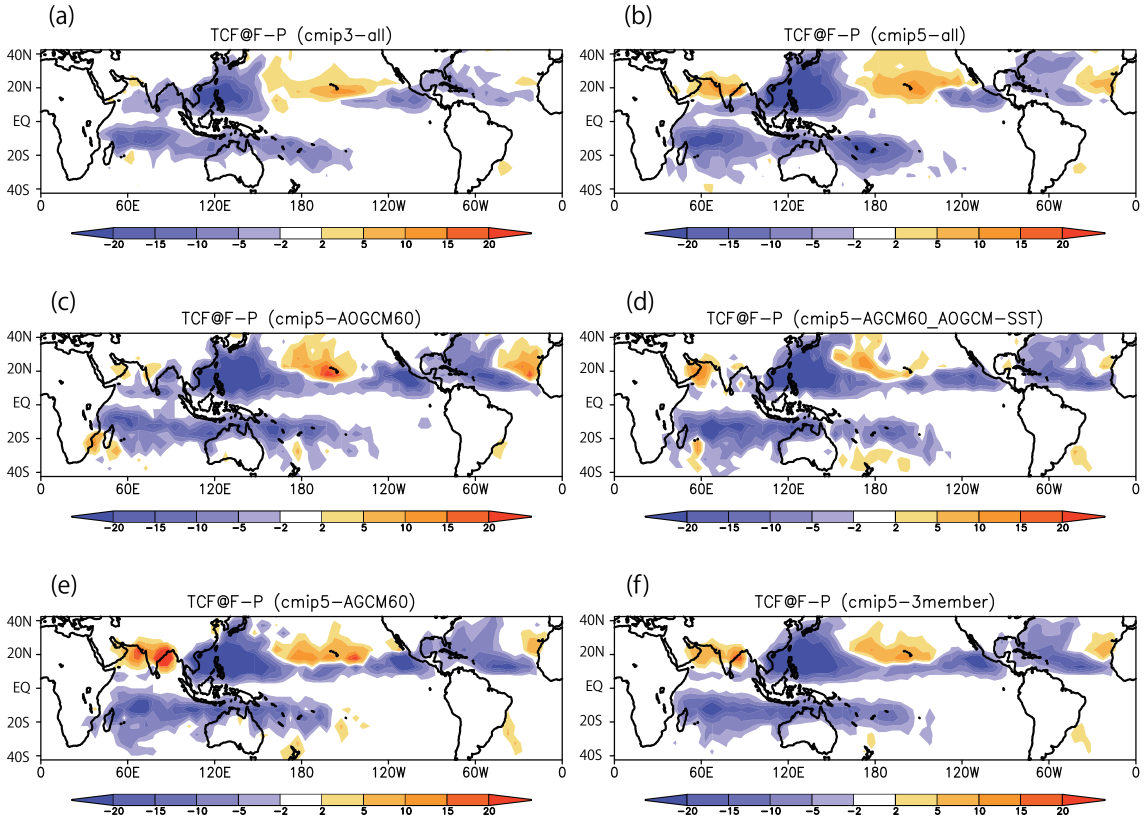

Figure S4: (a) Same as Fig. 3a but for all TCs in MRI-AGCM3.2 experiments under CMIP3-A1B scenario, 13 members (3 convection schemes × 4 future SST patterns = 12 members in 60km-AGCM, and 1 member in 20km-AGCM) ensemble mean (unit numbers/yr). (b) Same as (a) but for MRI-AGCM3.2 experiments under CMIP5-RCP8.5 scenario, 7 members (3 members with different convection schemes in 60km-AGCM, and 4 members with different future SST patterns in 20km-AGCM) ensemble mean. (c) 60km-AOGCM, (d) 60km-AGCM with AOGCM-SST, (e) 60km-AGCM with observed HadISST, and (f) ensemble mean of (c)-(e) is also shown. All plots and maps are generated by GrADS version 2.0.2 (http://cola.gmu.edu/grads/).
